# Annotations

**Published:** 1894-02-10

**Authors:** 


### ANNOTATIONS.

### Toothbrush Drill.

MANY physiological improvements have been made in the habits of the people since the time when that Scotch maidservant, of whom Dean Ramsay tells us, rushed breathlessly to her mistress to tell her that Mr. Smith, who was going to dine with the family, must be a tremendous eater, because she had found him in the bedroom with a little brush in his hand "sharpening his teeth." The use of a toothbrush is happily now a common experience, even among the class of maidservants. Mr. Denison Pedley, F.R.C.S., dental surgeon to the Evelina Hospital, in his report on the examination of 661 boys of the Industrial School at Feltham, recommends, among other things, a "toothbrush drill" for the boys at the close of the last meal of every day. This suggestion will appear to some people to be of the nature of a counsel of perfection. But it is by no means so, as certain teeth statistics quoted by Mr. Pedley abundantly show. Of these 661 boys, more than three-fourths had decayed teeth at one or other stage. Among the 500 odd boys whose teeth were not perfect, there were 1,744 defective grinders, and 741 of these were permanently diseased, and required the considerable operation of filling. Mr. Pedley, who speaks from the doctors as well as from the dentists' point of view, insists upon the disabilities under which children labour whose teeth are defective. Not only are such children liable to the pains and sufferings of toothache, but their powers of digestion and nutrition are bound to be impaired by the imperfect mastication which defective teeth necessitate. Moreover, diseased teeth produce an active poison, the poison of decomposed bone, in the mouth, and thus render the breath offensive, and at the same time the food less wholesome. The question is much more serious than some of us are apt to suppose, and a daily "tooth brush drill" at the close of the evening meal would be an excellent regulation for all sorts and conditions of children.

### The International Sanitary Conference.

THE wolves and the lambs of differing nationalities may very well "dwell" together in amity when the spectre of cholera hovers in the near distance ready to involve both in a common destruction. The International Conference at Paris will, we sincerely hope, be animated by one purpose only—the purpose of a common defence. If this is to be so, every nationality must be prepared to see itself, not with its own eyes only, but with the eyes of other nationalities. Dr. E. Klein, for example, seems to see France and her "cholera" ways in a very different light from that in which she is seen by her own specialists. We may certainly assume that French sanitarians and cholera specialists are quite as anxious for the extinction of cholera as is Dr. Klein. Nevertheless, it appears to us that Dr. Klein, in his recent letter to the *Times*, has really hit two very serious blots in the French system of dealing with cholera in an epidemic or sub-epidemic form. The French plenipotentiary for the conference proposes to direct attention to the far East, but principally to Mecca, as the great *fons et origo* of the cholera epidemics which devastate Europe. That, says Dr. Klein, would be closing up one floodgate whilst leaving another and even larger one wide open. France, he maintains, imports her own cholera; and when she has imported it, she makes no attempt to extinguish it until its blaze is so great as to illuminate the whole sky. Tonkin, and not Mecca only, is a principal source of French cholera. Two French epidemics, within the last ten years, appear to have had their origin at Tonkin—the first in 1884-86, the second, which so seriously alarmed Paris and this country, in 1892. But Dr. Klein calls attention to another factor in French sanitation more serious even than this. French notification, he says, in effect, is a farce. In Germany, as in England, every single case of cholera, wherever it may occur, is singly and separately notified. In France, notification is not compulsory until a cholera "foyer" or "focus" is established in any particular commune. But what is a "focus" of cholera? Is it one case, or is it a thousand? The decision of this question is left to the local authorities. We sincerely hope that red tape, professional *amour propre*, and even patriotism may be kept in abeyance, and that the higher brotherhood of science and common defence may constitute a union which will be able to raise up an impregnable barrier against the influx of cholera into Europe from any quarter whatsoever.

### Shall London Have a University?

THE Gresham Commission appointed to consider the question of a Teaching University for London has issued a summary of its recommendations. These are of interest to members of the medical profession, first as good citizens, and secondly as specialists in the medical art. London is the commercial centre of the world, and like all commercial centres, ancient or modern, it has drawn together the most cosmopolitan population in the world. It is eminently adapted for a seat of learning, and a seat of learning of the very broadest and most universal order. But London has never had a university. A degree granting body is no more a university than a board of military examiners is an army. London is unique in the opportunities it offers, and in the capable men it draws to it as a teaching centre. A teaching centre it must become, and no longer must it permit its extraordinary talents and opportunities to run to waste. The scheme of the Gresham Commissioners gives enviable pre-eminence to science and medicine in the proposed university. This is gratifying, no doubt, to us as a profession, and recognises the actual facts of the moment, but we question its wisdom, or the greatness of the ideal thus brought into view. We do not want a medical university, or a theological, or a legal, or an academical, but a university simply; and though no doubt the natural homes of letters and theology are at the present moment at the older universities, whilst the natural homes of science and medicine are in London, we yet hope that the natural home of everything that is human and liberal may be found in London in the near future. A university of one, or even of two faculties is very much to be deprecated. If we are to create a university of and for London and the Empire let it be imperial, and even universal in conception, and in practice a model and an object lesson for the whole world.

